# Human Antibodies against Herpes Simplex Virus 2 Glycoprotein G Do Not Neutralize but Mediate Antibody-Dependent Cellular Cytotoxicity

**DOI:** 10.3390/antib13020040

**Published:** 2024-05-11

**Authors:** Jan-Åke Liljeqvist, Karin Önnheim, Petra Tunbäck, Kristina Eriksson, Staffan Görander, Malin Bäckström, Tomas Bergström

**Affiliations:** 1 Department of Infectious Diseases, Institute of Biomedicine, 413 90 Gothenburg, Sweden; karin.onnheim@gu.se (K.Ö.); staffan.gorander@gu.se (S.G.); tomas.bergstrom@microbio.gu.se (T.B.); 2 Department of Clinical Microbiology, Region Västra Götaland, Sahlgrenska University Hospital, 413 46 Gothenburg, Sweden; 3 Department of Dermatology and Venereology, Institute of Clinical Sciences, University of Gothenburg, 413 45 Gothenburg, Sweden; petra.tunback@derm.gu.se; 4 Department of Rheumatology and Inflammation Research, Institute of Medicine, Sahlgrenska Academy, University of Gothenburg, 413 90 Gothenburg, Sweden; kristina.eriksson@microbio.gu.se; 5 Mammalian Protein Expression Core Facility, The Sahlgrenska Academy, University of Gothenburg, 413 90 Gothenburg, Sweden; malin.backstrom@gu.se

**Keywords:** concentrations of anti-gD-2 and anti-EXCT4-mgG-2 antibodies, herpes simplex virus 1 and 2 infection, neutralization activity, ADCC, CDC

## Abstract

Herpes simplex virus 2 (HSV-2) is a sexually transmitted infection affecting 491 million individuals globally. Consequently, there is a great need for both prophylactic and therapeutic vaccines. Unfortunately, several vaccine clinical trials, primarily employing the glycoprotein D of HSV-2 (gD-2), have failed. The immune protection conferred by human anti-HSV-2 antibodies in genital infection and disease remains elusive. It is well-known that gD-2 elicits cross-reactive neutralizing antibodies, i.e., anti-gD-2 antibodies recognize gD in HSV-1 (gD-1). In contrast, anti-glycoprotein G in HSV-2 (mgG-2) antibodies are exclusively type-specific for HSV-2. In this study, truncated versions of gD-2 and mgG-2 were recombinantly produced in mammalian cells and used for the purification of anti-gD-2 and anti-mgG-2 antibodies from the serum of five HSV-2-infected subjects, creating a pool of purified antibodies. These antibody pools were utilized as standards together with purified mgG-2 and gD-2 antigens in ELISA to quantitatively estimate and compare the levels of cross-reactive anti-gD-1 and anti-gD-2 antibodies, as well as anti-mgG-2 antibodies in sera from HSV-1+2-, HSV-2-, and HSV-1-infected subjects. The median concentration of anti-mgG-2 antibodies was five times lower in HSV-1+2-infected subjects as compared with cross-reactive anti-gD-1 and anti-gD-2 antibodies, and three times lower in HSV-2 infected subjects as compared with anti-gD-2 antibodies. The pool of purified anti-gD-2 antibodies presented neutralization activity at low concentrations, while the pool of purified anti-mgG-2 antibodies did not. Instead, these anti-mgG-2 antibodies mediated antibody-dependent cellular cytotoxicity (ADCC) by human granulocytes, monocytes, and NK-cells, but displayed no complement-dependent cytotoxicity. These findings indicate that antibodies to mgG-2 in HSV-2-infected subjects are present at low concentrations but mediate the killing of infected cells via ADCC rather than by neutralizing free viral particles. We, and others, speculate that Fc-receptor mediated antibody functions such as ADCC following HSV-2 vaccination may serve as a better marker of protection correlate instead of neutralizing activity. In an mgG-2 therapeutic vaccine, our findings of low levels of anti-mgG-2 antibodies in HSV-2-infected subjects may suggest an opportunity to enhance the immune responses against mgG-2. In a prophylactic HSV-2 mgG-2 vaccine, a possible interference in cross-reactive immune responses in already infected HSV-1 subjects can be circumvented.

## 1. Introduction

Herpes simplex virus 2 (HSV-2) infects the genital mucosa and establishes a life-long infection in the sensory ganglia. Following a primary infection, HSV-2 may reactivate resulting in genital lesions or more commonly asymptomatic shedding of the virus. HSV-2 is wide-spread, with an estimated 491.5 million people aged 15–49 years infected globally, giving a worldwide prevalence of 13.2% in 2016 [1]. The same study estimated the annual incidence to be 23.9 million infections. HSV-2 infection also lead to recurrent meningitis, severe neonatal infection, and significantly increases the risk of acquiring HIV [2]. Given this epidemiological situation there is a need for the development of both therapeutic and prophylactic vaccines. However, several clinical trials have yielded discouraging results.

The primary target in these trials has been HSV-2 glycoprotein D (gD-2) due to its essential role for cell entry and the ability to induce neutralizing antibodies in HSV-infected subjects. For instance, Chiron’s adjuvanted gB-2/gD-2 prophylactic vaccine showed high levels of neutralizing antibodies but had an overall vaccine efficacy of only 9% [3]. GlaxoSmithKline (GSK) also tested an adjuvanted gD-2 in a prophylactic clinical vaccine trial in HSV-1- and HSV-2-negative women, which showed no protection against HSV-2 infection but did provide partial protection against HSV-1-induced disease and infection [4]. This protection correlated with the level of anti-gD-2 antibodies in ELISA but not with the cellular responses [5]. Additionally, serum from gD-2-vaccinated subjects also showed neutralizing activity against HSV-1 [6,7,8].

After HSV-1 and HSV-2 infection, antibodies are most frequently elicited against envelope proteins, followed by regulatory, tegument, and capsid proteins [9]. While anti-gD-2 monoclonals and human anti-gD-2 antibodies can cross-react and bind to HSV-1 gD-1 antigen, neutralizing HSV-1 [6,10,11,12,13], studies by Marsden et al. [14] and Liljeqvist et al. [15] identified a linear HSV-2 type-specific immunodominant stretch of 23 amino acids in the membrane bound portion of the envelope glycoprotein G of HSV-2 (mgG-2). This region contains both human and mouse monoclonal antibody epitopes and is widely used as an antigen for detecting anti-mgG-2 antibodies as a marker of an HSV-2 infection.

In a recent report, the antibody profiles were investigated after vaccination with an HSV-2 replication-defect vaccine HSV529 in HSV-1- and HSV-2-negative vaccine recipients and compared with the antibody responses in naturally HSV-2-infected subjects. Using a random peptide display library and serum antibodies, the two most enriched epitopes were located within the defined immunodominant epitope region of mgG-2, both after vaccination and after natural HSV-2 infection [16].

In an effort to produce an HSV-2 vaccine we have shown that mgG-2 together with adjuvant induced protection against genital and neurological disease in a mouse vaccination genital challenge model where anti-mgG-2 antibodies presented antibody-dependent cellular cytotoxicity (ADCC), and complement-dependent cytotoxicity (CDC) [17]. A recombinantly produced truncated version of the mgG-2 (EXCT4-mgG-2) also induced protection in the mouse vaccination model with different adjuvants [18]. However, although promising results have been described for several animal vaccination models, the results have only been partially predictive of the outcome in clinical trials.

We recently described that mgG-2 promotes virus release from the surface of infected cells by interaction with glycosaminoglycan mimicking oligosaccharides [19]. The function of anti-EXCT4-mgG-2 antibodies in human HSV-2 infection remains unknown. In this study, we first estimated the concentrations of cross-reactive anti-gD-1, anti-gD-2, and anti-mgG-2 antibodies in HSV-1+2-, HSV-2-, and HSV-1-infected subjects. Second, we estimated the levels of neutralizing activity of pools of purified anti-gD-2 and anti-mgG-2 antibodies, and finally, we evaluated whether purified human anti-EXCT4-mgG-2 antibodies present ADCC and/or CDC activity.

## 2. Material and Methods

### 2.1. Sera from Symptomatic HSV-1+2-Infected and Symptomatic HSV-2-Infected Subjects

Subjects infected with HSV-1+2 and HSV-2 showing genital symptoms were recruited from the sexually transmitted disease (STD) clinic at the Sahlgrenska University Hospital. To classify serostatus, all serum samples underwent analysis using an in-house *Helix pomatia* lectin purified mgG-2 (full-length protein produced from BHK-21-infected cells) in ELISA (HSV-2-specific) [15], along with commercially available HerpeSelect1 (HSV-1-specific, gG1-antigen) and HerpeSelect2 (HSV-2-specific, gG2-antigen) ELISA IgG (Focus Technologies, Cypress, CA, USA), and chemiluminescent immunoassay (CLIA) LiaisonXL HSV1 IgG (gG1-antigen) and HSV2 IgG (gG2-antigen) (Diasorin S.p.A, Saluggia, Italy). The characterization of 33 HSV-1+2- and 37 HSV-2-infected subjects, presenting 3 - >10 recurrences/year, is described in Table 1. In the HSV-1+2 cohort, HSV-2 infection was confirmed by PCR on clinical lesions for 16 subjects. In the HSV-2 cohort, HSV-2 infection was confirmed by PCR on clinical lesions for eight persons. The subjects presented genital symptoms ≥1 year. Therefore, no primary infections were included.

### 2.2. Sera from HSV-1-Infected and HSV-Negative Subjects

Ten consecutive samples were collected from the pool of sera submitted to the Clinical Virological Laboratory at Sahlgrenska University Hospital for routine testing (Table 1). Samples were selected based on an end-point titer of ≥3200 to an in-house HSV-1/2 cross-reactive sodium deoxycholate-solubilized HSV-1-infected membrane preparation in ELISA [20]. Additionally, serological diagnosis of HSV-1 infection was confirmed by HerpeSelect1 ELISA IgG, and by CLIA LiaisonXL HSV1 IgG. Sera from the HSV-1-infected subjects were negative to *Helix pomatia* lectin purified mgG-2, negative in HerpeSelect2 ELISA IgG, and negative in LiaisonXL HSV2 IgG. Information on clinical symptoms of the HSV-1-infection was not available, but none of the sera were analyzed specifically because of symptoms associated with genital HSV infection. Sera from three HSV-1- and HSV-2-negative subjects were included as controls and were negative to the HSV-1/2 in-house antigens and in the HSV-1 and HSV-2 type-specific assays.

### 2.3. Production of EXCT4-mgG-2 and Coupling to an Immunosorbent Column

The EXCT4-mgG-2 construct codes for the entire extracellular region (EX) including residues alanine_345_–aspartic acid_649_, the entire cytoplasmic (C) region including residues alanine_671_–aspartic acid_698_, and four residues in the transmembrane region (T4) including residues valine_667_–alanine_670_ (HSV-2 strain 333, GenBank accession number LS480640). EXCT4-mgG-2 was expressed and purified as described earlier [18]. Briefly, the coding sequence was subcloned into pEE12.4 using Chinese hamster ovary (CHO)-K1 cells provided with the GS expression system (Lonza, Cambridge, United Kingdom). The protein was purified over an anion exchange column Hiprep16/10QFF (Pharmacia, Uppsala, Sweden) followed by a Sephacryl S-400 high resolution 16/60 gel filtration column (Pharmacia, Uppsala, Sweden). The target protein was detected with an apparent molecular weight of 80–85 kDa in Western blot and the purity was estimated to be ≥80% based on SDS-PAGE and Imperial blue staining [18]. The antigen was coupled to an epoxy-activated Sepharose 6B column (Cytiva, Life Sciences, Uppsala, Sweden) as described [15].

### 2.4. Production of gD-2 and Coupling to an Immunosorbent Column

The entire extracellular region of gD-2 (amino acids 26-342) was produced by recombinant technique in CHO-K1 cells as described [21], kindly provided by A.M. Harandi. The protein was purified using a HiTrap Chelating HP column (GE Healthcare, Freiburg, Germany). The target protein was detected with an apparent molecular weight of 42 kDa in Western blot and the purity was estimated to be 95% based on SDS-PAGE and Imperial blue staining. The gD-2 antigen was coupled to a cyanogen bromide-activated Sepharose 4B gel according to manufacturer’s instructions (Cytiva Life Sciences, Uppsala, Sweden).

### 2.5. Production of Pools of Purified Human Antibodies Used for Standard Curves and for Functional Assays

To create a standard for polyclonal antibody reactivity to EXCT4-mgG-2 and to gD-2 antigens, five sera from symptomatically HSV-2-infected subjects were used. To increase the yield of antibodies and include different antibody specificities, sera were selected presenting high titers of anti-mgG-2 antibodies to the in-house *Helix pomatia* lectin purified mgG-2, and high titers of anti-gD-2 antibodies to an in-house HSV-1/2 cross-reactive sodium deoxycholate-solubilized HSV-1-infected membrane preparation (titers ≥ 6400). The serum samples were classified as HSV-2 positive and HSV-1 negative by *Helix pomatia* lectin purified mgG-2 ELISA, HerpeSelect1 IgG, HerpeSelect2 IgG ELISA (Focus Technologies, Cypress, CA, USA), and by CLIA LiaisonXL HSV1 IgG and HSV2 IgG (Diasorin S.p.A, Saluggia, Italy). The serum samples (1.2 mL each) were pooled and diluted 1:4 in Tris-buffered saline (TBS) to a total volume of 24 mL. The EXCT4-mgG-2 and the gD-2 affinity columns were equilibrated with 30 mL TBS, and 6 mL of diluted serum samples were applied to each of the columns and recirculated for 1 h. After washing, antibodies were eluted with 0.1 M glycine-HCl pH 2.8 and neutralized with 1 M Tris-HCl pH 8.0. Purified samples were thereafter pooled. IgG-antibody concentrations were measured with a Human IgG ELISA kit (Ramcom A/S, Stockholm, Sweden). Flow-through from the EXCT4-mgG-2 immunosorbent column, used for purification of anti-EXCT4-mgG-2 antibodies, was unreactive to in-house *Helix pomatia* lectin purified mgG-2, and flow-through from the gD-2 immunosorbent column, used for purification of anti-gD-2 antibodies, was negative for in-house HSV-1/2 cross-reactive sodium deoxycholate-solubilized HSV-1-infected membrane preparation in ELISA (OD values < 0.2), suggesting a complete acquisition of antigen-specific antibodies.

### 2.6. The Specificity of Pools of Purified Antibodies

The reactivity of pools of purified anti-EXCT4-mgG-2 and anti-gD-2 antibodies were evaluated against several in-house antigens and in the commercial HSV-1 and HSV-2 type-specific assays. The EXCT4-mgG-2 and gD-2 antigens were produced as described. A 128-amino-acid-long peptide of mgG-2 was synthesized (LifeTein, Somerset, NJ, USA, 96% purity), including alanine_550_–aspartic acid_649_ and the entire cytoplasmic region including residues alanine_671_–aspartic acid_698_. The peptide covered the immunodominant region in human and monoclonal antibody epitopes [14,15]. Full-length glycoprotein B-2 (gB-2) was produced from GMK-AH1 HSV-2-infected cells using an immunosorbent column containing anti-gB-antibodies, and full-length glycoprotein C-2 (gC-2) was produced from HSV-2-infected BHK-21-cells using an immunosorbent column containing polyclonal rabbit anti-gC HSV antibodies [22]. Additionally, the extracellular region of gC-2 was recombinantly produced in HEK-293-cells (GenScript, Piscataway, NJ, USA).

Purified antigens were coated onto Maxisorp microtiter plates (Nalge Nunc International Corporation, Rochester, NY, USA) at a concentration of 1.0 µg/mL in carbonate buffer (pH 9.6) at 4 °C. The in-house cross-reactive HSV-1/2 antigen was coated at a 1:1000 dilution, and the mgG-2 peptide at a concentration of 5 µg/mL. Prior to use, the plates were washed and blocked with 2% skim milk in phosphate-buffered saline (PBS) for 30 min at 37 °C.

Pooled purified anti-EXCT4-mgG-2 and anti-gD-2 antibodies were evaluated at a 1:100 dilution resulting in a final concentration of 5 µg/mL. An HSV-negative serum was used as control, defining the cut-off as the mean of four wells plus two standard deviations (SD).

For the evaluation of cross-reactivity of IgG antibodies in other herpes viruses to the EXCT4-mgG-2 and gD-2 antigens, seven HSV-negative sera containing IgG antibodies to cytomegalovirus, Epstein-Barr virus (Alinity, Abbot Laboratories, Chicago, IL, USA), human herpes virus 6 (Vidia spol. s r.o., Vestec, Czech Republic), and recombinantly produced glycoprotein E of varicella zoster virus in an in-house ELISA [23], were selected for evaluation. These sera were tested at a 1:100 dilution in ELISA, and the cut-off was defined using the same HSV-negative control serum as described for the purified antigens above.

### 2.7. Measurement of Antibody Concentration in Serum in ELISA

After optimizing serum reactivity to purified EXCT4-mgG-2 and gD-2 antigens of HSV-1+2-, HSV-2-, and HSV-1-infected subjects, as well as HSV-negative subjects, sera were screened in duplicate at a 1:1600 dilution for anti-EXCT4-mgG-2 antibodies and at a 1:3200 dilution for anti-gD-2 antibodies. Purified antibodies were diluted in PBS containing 1% skim milk and 0.05% Tween-20.

From the endpoint titer created by the standard curves, an optical density (OD) interval of ±0.3 was defined from the inflection point to ensure linearity between the OD value and the concentration. When necessary for individual serum samples, the dilution was adjusted so the OD value was within the defined OD interval.

Standards of the pools of purified antibodies were included on each plate in two-fold dilutions. Each serum was evaluated simultaneously for both antigens. The plates were incubated for 1.5 h at 37 °C. After washing, alkaline phosphates labeled goat anti-human IgG (Jackson ImmunoResearch Laboratories, West Grove, PA, USA) at a 1:1000 dilution served as the conjugate, and P5994-25T tablets (Sigma-Aldrich, Stockholm, Sweden) dissolved in a DEA buffer served as the substrate. OD values were read in a spectrophotometer at 405 nm, and the concentrations of antibodies were calculated from the standard curves. The cut-off for positive samples was defined as 2 µg/mL. Intra- and inter-assay coefficients of variation in the ELISA were calculated using two sera from HSV-2-infected subjects in five positions on the same Maxisorp microtiter plate and on three separate plates at different occasions.

### 2.8. Neutralization Assay without or with Complement

GMK-AH1 cells were employed for the viral plaque reduction assay. Pools of purified anti-EXCT4-mgG-2 or anti-gD-2 antibodies, with two-fold dilutions of antibodies at a start dilution of 10 µg/mL, were evaluated without or with complement. An HSV-negative serum was used as complement source at a final concentration of 2.5%. Antibody samples were mixed with 150 HSV-2 (strain 333) plaque-forming units and incubated for 1 h at 37 °C and transferred to the cells. A methylcellulose overlay was applied, and the plaques were counted after 72 h. An inactivated HSV-negative serum and an inactivated HSV-2-positive serum were used as controls at 1:100 dilution. The lowest concentrations of antibodies which reduced the number of plaques by 50% (NT_50_) were calculated based on the inactivated HSV-negative serum control.

### 2.9. Antibody-Dependent Cellular Cytotoxicity (ADCC) and Complement-Dependent Cytotoxicity (CDC)

Blood granulocytes were purified from buffy coats from a blood donor using dextran sedimentation and a Ficoll–Paque gradient centrifugation, resulting in 93–97% purity [24]. Erythrocytes were disrupted by hypotonic lysis, and granulocytes were washed and resuspended in a Krebs–Ringer solution with glucose and kept on ice. Monocytes and NK-cells were prepared from peripheral blood mononuclear cells (PBMC) using the BD Imag™ Human Monocyte and NK Cell Enrichment Sets (BD Biosciences, Stockholm, Sweden).

BHK-21-cells were infected with the HSV-2 strain 333 at a multiplicity of infection of four and used as target cells for both ADCC and CDC. After an 18 h infection, cells were dispersed using EDTA-buffer supplemented with 0.025% trypsin and washed four times in TBS supplemented with 2% inactivated fetal calf serum (IFC). After washing, cells were diluted in Eagle’s minimal essential medium supplemented with 10% IFC (EMEM). Cell counts were adjusted to 10^6^ cells/mL and labeled with Calcein AM (Thermo Fisher Scientific, Uppsala, Sweden) [25]. Cells were washed in EMEM and adjusted to 10^5^ cells/mL for ADCC and to 2 × 10^5^ cells/mL for CDC. An HSV-2-positive serum, an HSV-negative serum, and a pool of purified anti-EXCT4-mgG-2 antibodies were diluted in EMEM.

For the ADCC-assay granulocytes, monocytes, NK-cells, and PBMC were used as effector cells, with an HSV-2-positive serum as the control. Assays were performed in a U-bottom 96-well microtiter plate (Nunc, Roskilde, Denmark). Serum and the pool of purified anti-EXCT4-mgG-2 antibodies, which was used for standards in the ELISA and for the neutralization assay, were tested in duplicate, where each well contained 50 μL target cells mixed with 50 μL diluted HSV-2-positive serum (positive control) or anti-EXCT4-mgG-2 antibodies. Optimal effector:target (E:T) ratios were set to 100:1 for granulocytes, 50:1 for PBMC and monocytes, and 5:1 for NK-cells. The cut-off for positive signal for pool 1 of purified anti-EXCT4-mgG-2 antibodies was set to 5% specific cytotoxicity based on the mean value of the fluorescence intensity from E:T wells + 2 SD.

Fresh PBMCs were used in the preparation of the monocytes and NK-cells and derived from the same donor. Two different blood donors were used for preparation of granulocytes. Each run included four wells containing only E:T cells and medium, and three wells containing target cells and 2% Triton-100 (max release). Cells and antibodies were incubated at 37° C in 5% CO_2_ for 6 h. 

For the CDC assay, the target cells, the HSV-2-positive serum, and purified anti-EXCT4-mgG-2 antibodies, were mixed as described above. Three wells with target cells plus complement and three wells with only target cells for maximum release were included. Plates were incubated for 1 h in 5% CO_2_ followed by addition of 10% human HSV-negative serum as source of complement and finally incubated for another 2 h. Additionally, *Helix pomatia* lectin purified anti-mgG-2 antibodies [15] from five HSV-2-positive sera were used. 

For both ADCC and CDC assays, 75 μL of the supernatants from each well were transferred to a 96-well flat bottom microtiter plate (Nunc, Roskilde, Denmark). Fluorescence intensity was measured using FLUOstar Omega (BMG Labtech, Ortenberg, Germany), with excitation of 485 nm and emission of 520 nm. The mean values from two wells (serum and antibodies), from three wells (maximum release), and from four wells (E:T) were used for calculation of ADCC. In a similar way, the mean value from two wells (serum and antibodies), and from three wells (target cells plus complement and maximum release) were used for calculation of CDC.

For granulocytes, monocytes, NK-cells, and for PBMC, three independent experiments were performed. Specific cytotoxicity for ADCC was calculated as [(test release − ET-release)/(max release − ET-release)], and for CDC as [(test release − target + complement release)/(max release − target + complement release)].

## 3. Statistics

The distribution of the antibody concentrations in sera from HSV-1+2- and HSV-2-infected subjects was tested using the D’Agostino–Pearson and the Shapiro–Wilk normality tests (GraphPad Prism, Gothenburg University, Sweden). As the concentrations of anti-EXCT4-mgG-2 and anti-gD-2 antibodies in the HSV-2-infected cohort were not normally distributed, the nonparametric two-tailed Mann–Whitney test was used for comparison. A *p* value of <0.05 was considered significant.

## 4. Results

### 4.1. Specificity of Purified anti-EXCT4-mgG-2 and anti-gD-2 Antibodies

Recombinant EXCT4-mgG-2 and gD-2 antigens were produced in CHO-K1 cells and coupled to an immunosorbent column for purification of antibodies from the serum of five HSV-2-infected subjects. Specificity of pooled purified antibodies and seven HSV-negative sera containing IgG to other herpes viruses were evaluated to EXCT4-mgG-2 and gD-2 antigens with in-house ELISAs and in the commercial type-specific assays (Table 2).

The pool of anti-EXCT4-mgG-2 antibodies demonstrated specific reactivity to *Helix pomatia* lectin purified mgG-2 antigen and to the mgG-2 peptide, positive in HerpeSelect2 IgG (Focus Technologies, Cypress, CA, USA), and positive in CLIA LiaisonXL HSV2 IgG (Diasorin S.p.A, Saluggia, Italy), while negative to an HSV-1/2 cross-reactive sodium deoxycholate-solubilized HSV-1-infected membrane preparation, negative in HerpeSelect1 IgG, negative in CLIA LiaisonXL HSV1 IgG, and negative to the gB-2 antigen and to two gC-2 antigens, as well as to the recombinantly produced gD-2 antigen (Table 2). As a positive control, the pool of anti-EXCT4-mgG-2 antibodies presented a mean OD value of 2.7 to purified EXCT4-mgG-2 antigen. Similar reactivity was observed to *Helix pomatia* lection purified mgG-2 antigen and to the mgG-2 peptide and classified as clearly positive with mean OD values between 2.5 and 3.0.

The pool of anti-gD-2 antibodies was reactive to the cross-reactive HSV-1 antigen, but negative in the commercial type-specific assays (gG1 and gG2 antigens), negative to *Helix pomatia* lectin purified mgG-2 antigen, negative to the mgG-2 peptide, and negative to the gB-2 and gC-2 antigens (Table 2). As a positive control, the pool of anti-gD-2 antibodies presented a mean OD value of 3.6 to purified gD-2 antigen.

The results obtained from testing seven HSV-negative sera, which contained IgG antibodies to cytomegalovirus, Epstein-Barr virus, human herpes virus 6, and varicella-zoster virus, showed OD values below 0.2 for specified antigens (Table 2). Based on these findings, it can be concluded that the purified anti-EXCT4-mgG-2 and anti-gD-2 antibodies exhibit high specificity. 

### 4.2. Concentrations of Antibodies in HSV-1+2-, HSV-2-, and HSV-1-Infected Subjects

The concentration of antibodies in clinical sera was measured using ELISA with the EXCT4-mgG-2 and gD-2 antigens, utilizing pools of purified anti-EXCT4-mgG-2 and anti-gD-2 antibodies for standard curves. Since EXCT4-mgG-2 is an HSV-2 type-specific antigen, the ELISA specifically detects anti-EXCT4-mgG-2 antibodies in HSV-1+2- and HSV-2-infected subjects. In contrast, as gD-2 is a cross-reactive antigen, the ELISA detects both cross-reactive anti-gD-1 (HSV-1) and anti-gD-2 (HSV-2) antibodies in HSV-1+2-infected subjects. In HSV-2-infected subjects anti-gD-2 antibodies were detected, while in HSV-1-infected subjects cross-reactive anti-gD-1 antibodies were detected.

**Figure 1:**
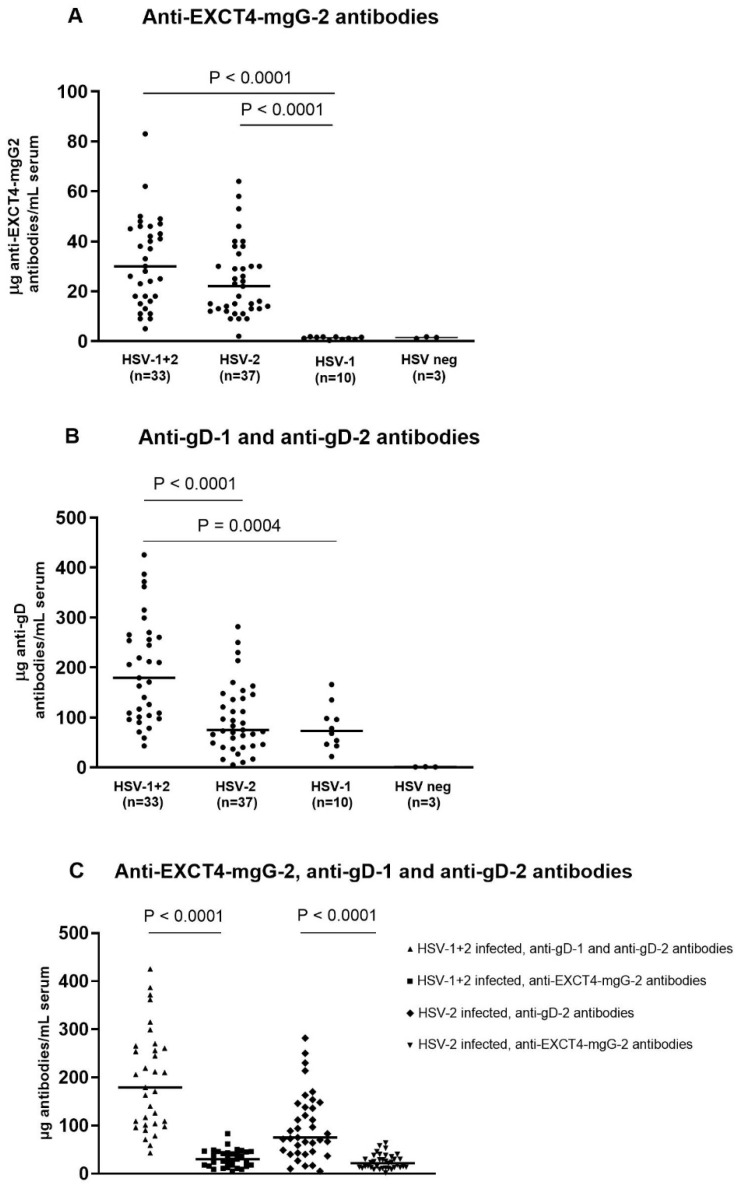
(**A**–**C**) Quantification of antibodies in clinical sera. Anti-EXCT4-mgG-2 antibodies, cross-reactive anti-gD-1 and gD-2 antibodies in sera of HSV-1+2-infected, HSV-2-infected, HSV-1-infected and HSV-negative subjects were evaluated using purified EXCT4-mgG-2 and gD-2 antigens in ELISA. Pools of purified anti-EXCT4-mgG-2 and anti-gD-2 antibodies with known concentrations were included for generation of standard curves. For statistical analyses the Mann–Whitney nonparametric test was used. The median values are marked with a horizontal line.

The median concentration of anti-EXCT4-mgG-2 antibodies in HSV-1+2-infected subjects (*n* = 33) was 30 µg/mL (range: 5 to 83), while for HSV-2-infected subjects (*n* = 37) it was 22 µg/mL (range: 2 to 64). A few sera from both HSV-1+2- and HSV-2-infected subjects had concentrations below 10 µg/mL. All HSV-1-positive and HSV-negative sera had concentrations below the cut-off of 2 µg/mL. Significant differences were observed between the concentrations of anti-EXCT4-mgG-2 antibodies in HSV-1+2- and HSV-2-infected subjects compared to HSV-1-infected subjects. Individual data points are shown in Figure 1A.

The median concentration of cross-reactive anti-gD-1 antibodies and anti-gD-2 antibodies in HSV-1+2-infected subjects (*n* = 33) was 179 µg/mL (range: 43 to 425). For HSV-2-infected subjects (*n* = 37), the median concentration of anti-gD-2 antibodies was 75 µg/mL (range: 5 to 282), while for cross-reactive anti-gD-1 antibodies in HSV-1-infected subjects it was 73 µg/mL (range: 22 to 166). The HSV-negative sera had concentrations below the cut-off of 2 µg/mL. There were highly statistically significant differences between the concentrations of cross-reactive anti-gD-1 and anti-gD-2 antibodies in HSV-1+2-infected subjects compared to HSV-2- and HSV-1-infected subjects. Individual data points are illustrated in Figure 1B.

The median concentration of anti-EXCT4-mgG-2 antibodies was 5.42 times lower compared to cross-reactive anti-gD-1 and anti-gD-2 antibodies in HSV-1+2-infected subjects, and 3.26 times lower in HSV-2-infected subjects. These differences were highly statistically significant (*p* < 0.0001), as depicted in Figure 1C.

Regarding assay variability, the mean intra-assay variation for measured concentrations was low for both anti-EXCT4-mgG-2 antibodies (7.6%) and anti-gD-2 antibodies (8.3%). The inter-assay variation was 10.3% for anti-EXCT4-mgG-2 antibodies and 9.0% for anti-gD-2 antibodies. These results indicate good assay reliability and reproducibility.

### 4.3. Neutralization Activity

The concentrations of pools of purified anti-EXCT4-mgG-2 and anti-gD-2 antibodies, which reduced the number of HSV-2 plaques by 50% without and with complement, are listed in Table 3. For the anti-EXCT4-mgG-2 antibodies no neutralization activity was observed (>10 µg/mL). The pool of anti-gD-2 antibodies presented neutralization activity at all concentrations evaluated both without and with complement.

### 4.4. ADCC

**Figure 2:**
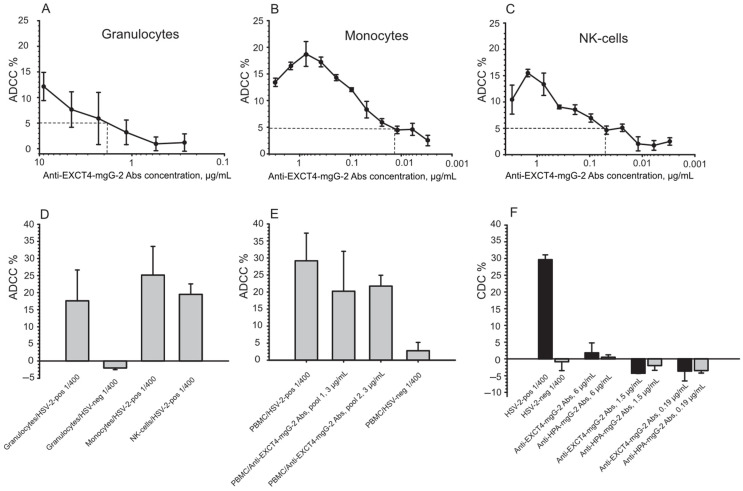
Antibody-dependent cellular cytotoxicity (ADCC) and complement-dependent cytotoxicity (CDC) of purified anti-EXCT4-mgG-2 antibodies (Abs). Pool 1 and pool 2 of anti-EXCT4-mgG-2 antibodies were each purified from five different HSV-2-infected sera. Pool 1 antibodies were evaluated for ADCC by human granulocytes (**A**), monocytes (**B**), and NK-cells (**C**). The dashed horizontal lines mark the cut-off which was defined as mean reactivity of target and effector cells without antibodies plus 2 SD. The dashed vertical lines in (**A**–**C**) define the cut-off concentrations of antibodies. An HSV-2-positive serum was diluted 1:400 and used as positive control for the different effector cells including an HSV-negative control serum for granulocytes (**D**). Total ADCC from peripheral blood mononuclear cells (PBMC), with an HSV-2-positive and with an HSV-negative serum, at 1:400 dilutions, and for comparison pool 1 and pool 2 of purified anti-EXTC4-mgG-2 antibodies, used at concentration of 3 µg/mL, were evaluated (**E**). Mean values from pool 1 were calculated from duplicate wells in two experiments (**A**–**D**) and from three experiments (**E**), except for pool 2 antibodies which was evaluated in one experiment. An HSV-2-positive serum, and pool 1 of purified anti-EXCT4-mgG-2 antibodies, and *Helix pomatia* lectin (HPA) purified anti-mgG-2 antibodies, in three concentrations, were evaluated in CDC in two experiments (**F**). The bars represent mean values +/− SD (**A**–**C**) and +SD (**D**–**F**).

Purified anti-EXCT4-mgG-2 antibodies were titrated in two-fold serial dilution, starting at 9 μg/mL for granulocytes, and at 3 μg/mL for monocytes and NK-cells. The cut-off for granulocytes was set to a concentration of 1.8 µg/mL of purified antibodies, the cut-off for monocytes was 0.015 µg/mL and 0.05 μg/mL for NK-cells (Figure 2A–C). For the highest antibody concentrations evaluated, lower ADCC was observed for monocytes and NK-cells, suggesting a pro-zone phenomenon.

An HSV-2-positive serum and an HSV-negative serum for granulocytes, were used at a 1:400 dilution and evaluated for the three effector cells. For granulocytes the mean value for the positive control was 18% (SD, 9), and no ADCC was observed with the HSV-negative control. The mean value of the positive control for monocytes was 25% (SD, 8.4), and for NK-cells 20% (SD, 3), (Figure 2D).

Using PBMC, an HSV-2-positive serum, used at a 1:400 dilution, presented 29% ADCC (SD, 8.1), (Figure 2E). Purified anti-EXCT4-mgG-2 antibodies, here designated as pool 1, presented 20% (SD, 12) ADCC, evaluated at a concentration of 3 µg/mL. As a control for variations of different specificities of purified anti-EXCT4-mgG-2 antibodies, five additional sera from HSV-2-infected subjects were included (designated as pool 2) and presented 22% (SD, 3.2) ADCC, (Figure 2E). We observed that the high standard deviation for pool 1 of purified EXCT4-mgG-2 antibodies, in Figure 2E, was because three independent experiments were included. Pool 2 of the purified anti-EXCT4-mgG-2 antibodies was used in a single experiment, and the standard deviation was lower. An HSV-negative serum presented 2.8% ADCC (SD, 2.5) (Figure 2E). 

### 4.5. CDC

The same HSV-2-positive serum used in the ADCC-assay was used in a 1:400 dilution and presented 30% specific cell lysis, Figure 2F. The cut-off for positive CDC was defined for ADCC as the mean value for target + complement +2 SD and set to 10%. Pool 1 of the purified anti-EXCT4-mgG-2 antibodies and *Helix pomatia* lectin purified anti-mgG-2 antibodies [15] were evaluated at the concentrations of 6 μg/mL, 1.5 μg/mL, and 0.19 μg/mL, and presented no CDC. The rationale to also use *Helix pomatia* lectin purified anti-mgG-2 antibodies was that mouse antibodies, after vaccination [17], presented CDC. Similarly, no CDC was detected, Figure 2F.

## 5. Discussion

The role of anti-HSV antibodies in immunity is often debated, with focus on whether HSV-2 antibodies can protect against HSV-2 infection. In a prophylactic clinical trial using adjuvanted gD-2 antigen (GSK), the vaccine did not protect against HSV-2-induced genital disease or infection [4]. However, anti-gD-2 antibodies, but not T cell responses, partially protected against genital HSV-1 disease and infection. These data challenge the notion that the cell-mediated immunity is most important in controlling genital HSV-1/2 disease and infection [26].

In this study, the EXCT4-mgG-2 and the gD-2 proteins were produced with recombinant technique in CHO-K1 cells and used for the purification of anti-EXCT4-mgG-2 and anti-gD-2 antibodies from symptomatically HSV-2-infected subjects. Using standard curves of a pool of purified antibodies, we were able to estimate the concentrations of specific antibodies in sera of HSV-infected and in HSV-negative subjects. A limitation with the study is that we only purified anti-gD-2 antibodies from HSV-2-infected subjects. We can therefore not estimate the levels of type-specific anti-gD-1 antibodies in HSV-1+2- and HSV-1-infected subjects. Such an estimation could be possible if also gD-1 antigen is produced, and anti-gD-1 antibodies are purified. Thus, in this study, the total amounts of anti-gD-1 antibodies in sera of HSV-1+2- and HSV-1-infected subjects are probably underestimated, (Figure 1B). 

The concentrations of anti-EXCT4-mgG-2 antibodies were significantly lower as compared with cross-reactive anti-gD-1 and anti-gD-2 antibodies in sera of HSV-1+2- and HSV-2-infected subjects. The low levels of anti-EXCT4-mgG-2 in serum were somewhat surprising as Wang et al. identified that the most enriched antibody-binding epitopes were localized within mgG-2 both in HSV-1- and HSV-2-negative subjects after HSV529 vaccination, as well as for HSV-2-naturally-infected subjects [16]. However, using peptides for epitope mapping is known to be biased towards linear epitopes [27]. The results may be explained by the fact that mgG-2 contains linear epitopes which are easily detected with peptide display libraries [16,28], using pepscan analysis with peptides coupled to a cellulose support [15], or with branched peptides [14]. Furthermore, antibody-binding to full-length mgG-2 was possible to block with peptides comprising the predicted epitopes in a competitive ELISA which support that the mapped epitopes are linear [15]. The presence of anti-mgG-2-antibodies has been used for several years as a type-specific marker of infection in serodiagnosis of HSV-2 infection. These observations were confirmed in this study, as sera of HSV-1-infected subjects presented no or negligible binding to EXCT4-mgG-2 antigen (Figure 1A).

Although mgG-2 is an HSV-2 type-specific protein and used in serodiagnosis of HSV-2 infection, this antigen has diagnostic limitations. For instance, false negative reactivity using a single mgG-2 based assay has been described in recurrent HSV-2-PCR-positive subjects [29]. These findings may at least partly be explained by the low concentrations of anti-EXCT4-mgG-2 antibodies. Approximately 10% of sera of HSV-1+2- and HSV-2-infected subjects presented concentrations of anti-EXCT4-mgG-2 antibodies below 10 µg/mL. Another possibility might be that not all anti-EXCT4-mgG-2 antibodies were captured to the immunosorbent column. In such a case, the pool of anti-EXCT4-mgG-2 antibodies, used as a standard for estimation of the amounts of antibodies in sera, could be affected. However, the flow-through from the column was negative to a different mgG-2 antigen (*Helix pomatia* lectin purified mgG-2), supporting a complete acquisition of antibodies.

We measured neutralization activity of purified antibodies as a functional aspect of anti-HSV-2 antibodies. Earlier studies have shown that HSV neutralization antibodies are mostly directed to gD, and in some extent to gB, for both symptomatic and for asymptomatic HSV-infected subjects [10,11]. A pool of anti-gD-2 antibodies presented neutralization activity both without and with complement at low concentrations (Table 3), while a pool of anti-EXCT4-mgG-2 antibodies presented no neutralization activity. Similarly, a lack of neutralization activity was shown in anti-mgG-2 monoclonal antibodies [30,31], in hyperimmune serum after vaccination with mgG-2 in mice [17], as well as in rabbit anti-mgG-2 hyperimmune serum [32].

We have described earlier that anti-mgG-2 antibodies, elicited after vaccination with *Helix pomatia* lectin purified full-length mgG-2, in a mouse genital challenge model present CDC and ADCC by macrophages and NK-cells. We show in this study for the first time that purified human anti-EXCT4-mgG-2 antibodies present ADCC by granulocytes, monocytes, NK-cells, and with PBMC. A limitation of the study is that the relative contribution of the anti-EXCT4-mgG-2 antibodies to the total ADCC activity cannot be estimated as the concentrations and specificities of other HSV-2 antibodies exerting ADCC in HSV-2 sera were not determined. Such an evaluation needs further studies, for instance, by a comparison of purified anti-gD-1, anti-gD-2 antibodies and antibodies to other HSV proteins from sera of HSV-1-, HSV-1+2-, and HSV-2-infected subjects. Depletion of serum in HSV-specific antibodies can also offer an available method.

For human IgG antibodies there are several receptors available for binding and activation via ADCC. Granulocytes can express CD64 (FcγRI), CD32A (FcγRIIA), and CD16B (FcγRIIIB); monocytes can express CD64 (FcγRI), CD32A/C (FcγRIIA/C), and CD16A (FcγRIIIA); and NK-cells can express CD32C (FcγRIIC) and CD16A (FcγRIIIA) [33]. Anti-EXCT4-mgG-2 antibodies may therefore exert a broad ADCC including several receptors on the effector cells. Although ADCC may be a potentially important mechanism for antibodies to control the HSV-2 infection, data on the role of such antibody response in human HSV-2 infection is sparse. In the clinical prophylactic HSV-2 vaccination trial, using adjuvanted gD-2 and gB-2 as immunization antigens (Chiron) [3], the poor efficacy may have partially been the result of low levels of antibodies capable of mediating ADCC [34]. Furthermore, the inability of anti-gD-2 antibodies to elicit ADCC may have contributed to the failure of the GSK’s clinical trial with adjuvanted gD-2 antigen [35]. Kohl et al. have described that antibodies exerting ADCC are important to protect neonates from disseminated HSV infection [36]. A novel vaccine strategy is the use of a gD-2 deleted replication-defective HSV-2 showing promising pre-clinical results in several studies. Interestingly, the vaccine induces weak neutralization antibody activity, but FcR-mediated antibody functions such as ADCC [37,38,39]. In addition, Kao et. al. have shown in a murine model that antibodies presenting ADCC protect neonates from both HSV-1 and HSV-2 [40]. The authors suggest that FcR-mediated antibody functions may be a more reliable correlate of immune protection than neutralizing antibodies in future vaccine trials.

No CDC activity was observed using a pool of anti-EXCT4-mgG-2 antibodies (Figure 2F). In contrast, in a mouse vaccination genital challenge model, anti-mgG-2 antibodies presented CDC activity [17]. We have not further investigated this discrepancy, but the source of complement may contribute. In the mouse studies we used guinea pig serum as a complement source, while in this study a human complement from an HSV-negative donor was used. Species differences in immunological effector functions may also be an explanation.

The cross-reactivity of monoclonal and human anti-gD-2 antibodies to gD-1 and HSV-1 is well known [6,10,11,12,13]. Our approach in the present study to use anti-gD-2 antigen in the ELISA to estimate the amounts of antibodies in sera from HSV-1+2- and HSV-1-infected subjects, was aimed to include cross-reactive anti-gD-1 antibodies in HSV-1-infected subjects. Several anti-gD-1 mouse monoclonal antibodies are type-common and bind to gD-2 antigen and neutralize HSV-2 [31,41,42]. In addition, a human monoclonal anti-gD-1 antibody neutralizes both HSV-1 and HSV-2 [43]. This cross-reaction is not surprising as the extra cellular regions of gD-1 (strain 17) and gD-2 (strain 333) present 89% amino acid identity (CLC Sequencer Viewer 8.0). We also confirmed in this study that there is a significant cross-reactivity of anti-gD-1 antibodies to the extracellular region of gD-2 of HSV-1 sera (Figure 1B).

Most subjects are HSV-1-infected in Western world countries and almost all in developing countries. HSV-1 usually infects early in life and the HSV-2 infects later after sexual debut. In a prophylactic HSV-2 vaccine, the inclusion of cross-reactive HSV-2 antigens to already HSV-1-infected subjects may introduce a potential obstacle. For instance, pre-existing antibodies elicited from earlier influenza infections or vaccinations can inhibit generation of antibody and B-cell proliferation to an updated influenza strain used in the annual influenza vaccination program [44]. In a randomized Phase 1 study using the replication-defective HSV-2 based HSV529 vaccine, most HSV-1- and HSV-2-negative subjects presented a ≥four-fold increase in neutralizing antibodies after three vaccinations, while HSV-1-positive subjects presented only a modest non-significant increase [45]. Such an obstacle may be circumvented using the HSV-2 type-specific mgG-2 as immunogen.

## 6. Conclusions

This study shows that low levels of anti-EXCT4-mgG-2 antibodies are detected in HSV-1+2- and HSV-2 infected subjects, a finding which may be favorable for a therapeutic vaccine aiming to enhance the antibody response to this protein. As the EXCT4-mgG-2 antigen is a type-specific HSV-2 protein, interference in pre-existing HSV-1 immune responses is less likely. The function of anti-EXCT4-mgG-2 antibodies exerting ADCC by granulocytes, monocytes, and NK-cells may be an important mechanism for both a prophylactic and therapeutic vaccine to combat the HSV-2 infection.

## Figures and Tables

**Table 1:** Characterization of the HSV cohorts for estimation of the concentrations of anti-EXCT4-mgG-2, anti-gD-1, and anti-gD-2 antibodies in sera. HSV-1+2- and HSV-2-infected subjects presented recurrent symptomatic genital HSV-2 disease. To classify serostatus, all serum samples were analyzed in ELISA using an HSV-1/2 cross-reactive sodium deoxycholate-solubilized HSV-1 infected membrane preparation, *Helix pomatia* lectin purified mgG-2 (HSV-2 specific), and by the commercially available type-specific HerpeSelect1 IgG, HerpeSelect2 IgG (Focus Technologies), and LiaisonXL HSV1 and HSV2 IgG (Diasorin) assays.

Cohorts	Median Age, Year (Range)	Number of Subjects (f, Female; m, Men)
HSV-1+2-infected	40 (22–69)	*n* = 33, (19 f, 14 m)
HSV-2-infected	36 (26–70)	*n* = 37, (18 f, 19 m)
HSV-1-infected	40 (16–65)	*n* = 10, (5 f, 5 m)
HSV-negative	26 (21–32)	*n* = 3, (2 f, 1 m)

**Table 2:** Reactivity of purified anti-EXCT4-mgG-2, anti-gD-2 antibodies (Abs), and HSV-negative sera. Pools of purified Abs, produced from serum of five HSV-2 infected subjects, and HSV-negative sera, containing IgG antibodies against other herpes viruses, were evaluated to different antigens. Reactivity was evaluated with in-house ELISA and in the commercial HSV-1 and HSV-2 type-specific assays (HerpeSelect and LiaisonXL). For the in-house ELISA, the cut-off was defined as the mean value of four wells + 2 SD using an HSV-negative serum, and reactivity was classified as: − negative (OD values < 0.3), + clearly positive (OD values between 2.5–3.0), and ++ highly positive (OD values ≥ 3.5). Results from the commercial assays were classified as negative or positive.

Antigens and Assays	HSV-Negative Sera ^a^	Anti-EXCT4-mgG-2 Abs	Anti-gD-2 Abs
HPA-mgG-2 ^b,c^	−	+	−
HSV-1 ^d^	−	−	++
HerpeSelect1 IgG (gG1 antigen, Focus)	neg	neg	neg
HerpeSelect2 IgG (gG2 antigen, Focus)	neg	pos	neg
LiaisonXL HSV1 (gG1 antigen, Diasorin)	neg	neg	neg
LiaisonXL HSV2 (gG2 antigen, Diasorin)	neg	pos	neg
EXCT4-mgG-2 (Rec ^e^)	−	+	−
gD-2 (Rec ^e^, EC ^f^)	−	−	++
gB-2 ^b^	ND	−	−
gC-2 ^b^	ND	−	−
gC-2 (Rec ^e^, EC ^f^, GenScript)	ND	−	−
mgG-2-peptide ^g^ (LifeTein)	ND	+	−

^a^ Seven HSV-negative sera presenting IgG antibodies to cytomegalovirus, Epstein Barr virus, human herpes virus 6 and varicella zoster virus, ^b^ full-length protein produced from virus-infected cells, ^c^ *Helix pomatia* lectin (HPA) purified mgG-2, ^d^ HSV-1/2 cross-reactive sodium deoxycholate-solubilized HSV-1-infected membrane preparation, ^e^ recombinantly produced, ^f^ Extra-cellular region, ^g^ 128-amino-acid-long peptide. ND, not determined.

**Table 3:** Viral plaque reduction assay in GMK-AH1 cells of pools of purified anti-EXCT4-mgG-2 and anti-gD-2 antibodies (Abs) from five symptomatically HSV-2-infected subjects. HSV-2 (150 plaque forming units, PFUs) was mixed with two-fold dilutions of antibodies with a start dilution of 10 µg/mL without (C^−^) or with complement (C^+^). An HSV-negative serum was used as complement source at a final concentration of 2.5%. The mean PFUs from two experiments without complement and from three experiments with complement are indicated. An inactivated HSV-negative control serum, a 1:100 dilution, presented a mean value of 75 PFUs without complement, and 70 PFUs with complement, and used for calculation of NT_50%_. An inactivated HSV-2-positive control serum, at a 1:100 dilution, presented no PFUs both without and with complement, and used as positive control.

Concentration of Abs, µg/mL	PFU with Anti-EXCT4-mgG-2 Abs		PFU with Anti-gD-2 Abs	
	C^−^	C^+^	C^−^	C^+^
10	65	60	0	0
5.0	69	66	0	0
2.5	70	72	0	0
1.25	68	75	0	0
0.63	72	70	0	0
0.31	75	73	0	2

## Data Availability

The original contributions presented in the study are included in the article, further inquiries can be directed to the corresponding author.

## Abbreviations

ADCC, antibody-dependent cellular cytotoxicity; CDC, complement-dependent cytotoxicity; EXCT4-mgG-2, truncated version of mgG-2
